# Maternal administration of probiotics promotes gut development in mouse offsprings

**DOI:** 10.1371/journal.pone.0237182

**Published:** 2020-08-07

**Authors:** Yueyue Yu, Jing Lu, Kaitlyn Oliphant, Nikhilesh Gupta, Katerina Claud, Lei Lu

**Affiliations:** Department of Pediatrics, Pritzker School of Medicine, The University of Chicago, Chicago, Illinois, United States of America; Universidade do Estado do Rio de Janeiro, BRAZIL

## Abstract

Necrotizing enterocolitis is the most common gastrointestinal disorder in premature neonates. This disease is characterized by massive epithelial necrosis, gut barrier dysfunction and improper mucosal defense development. Studies have shown that probiotic administration can decrease NEC incidence and mortality. The proposed mechanisms of probiotics for the prevention of NEC are: promotion of intestinal development; improved barrier function through decreased apoptosis and improved mucin production; decreased expression of proinflammatory cytokines IL6, IL8, and TNFα, and modulation of microbiota dysbiosis in preterm infants. However, reported sepsis in the immunocompromised preterm host has deterred routine prophylactic administration of probiotics in the neonatal intensive care unit. We hypothesize that maternal administration of probiotics to pregnant mouse dams can recapitulate the beneficial effects observed in neonates fed with probiotics directly. We exposed pregnant mice to the probiotics and monitored the changes in the developing intestines of the offspring. Pregnant mice were fed daily with the probiotics *Lactobacillus acidophilus* and *Bifidobacterium infantis* (LB) from embryonic day15 to 2-week-old postnatally. Intraperitoneal administration of IL-1β in the pups was used to model proinflammatory insults. Sera were collected at 2 weeks of age and evaluated for inflammatory cytokines by enzyme-linked-immunosorbent-assay and gut permeability by Fluorescein isothiocyanate-dextran tracer assay. Ileal tissues were collected for the evaluation of apoptosis and proliferation of the intestinal epithelium; as well as mucin and tight junction integrity at mucosal surface by immunofluorescent staining. We find that maternal LB exposure facilitated intestinal epithelial cell differentiation, prevented loss of mucin and preserved the intestinal integrity and barrier function and decreased serum levels of IL-1β, TNF-α and IL-6 in the preweaned offsprings. in LB exposed pups. We demonstrate that maternal probiotic supplementation promotes gut maturation in developing offspring. This is potentially a safe alternative therapy to induce intestinal maturation and prevent prematurity-associated neonatal disorders.

## Introduction

Necrotizing enterocolitis (NEC) is an inflammatory bowel necrosis that primarily afflicts preterm infants after the onset of enteral feeding. It is the most frequent gastrointestinal emergency of preterm infants, representing a major cause of morbidity and death in neonates [1]. This disease is characterized by massive epithelial necrosis, gut barrier dysfunction and improper mucosal defense development [2–5]. The pathophysiology of NEC remains poorly understood but the primary risk factors are prematurity and bacterial colonization [2–5]. Due to inadequate treatments and the absence of an effective preventative strategy, an estimated 20–40% of neonates with NEC require surgery [6], and 10–30% experience significant morbidities, including neurodevelopmental impairment, vision and hearing impairment, failure to thrive, feeding abnormalities, bowel stricture and short bowel syndrome. Thus, NEC continues to be an important health issue for neonates. Recent studies have indicated that prematurity remains the most consistent risk factor for developing NEC. The increased susceptibility of preterm infants to developing NEC is attributed to immature mucosal barrier development, increased susceptibility to inflammation and loss of epithelial integrity and abnormal intestinal microbiota patterns [1, 7].

Preterm infants have marked differences in the composition of their gut microbiota when compared to infants born at term, including a limited diversity and predominance of Proteobacteria [8]. Additionally, they are predisposed to a delayed acquisition of beneficial bacteria, particularly *Bifidobacterium*, *Lactobacillus* and *Bacteroides* [9]. A large number of randomized placebo-controlled clinical trials and cohort studies, in addition to research in animal models of NEC, have demonstrated a decrease in the incidence of NEC and all-cause mortality with administration of probiotics, as reviewed in [10]. However, concerns regarding the safety of probiotics and optimal dosing of the product in the immunocompromised premature neonate have limited the widespread adoption of routine clinical use of probiotics in preterm infants and alternative approaches are warranted [11]. Recently, Grev et al published their meta analysis review on maternal probiotic supplementation for prevention of morbidity and mortality in preterm infants. They compared probiotics administration vs placebo in 1) pregnant women at risk for preterm birth; 2) postpartum mothers who gave birth to preterm infants; and 3) probiotics to postpartum mothers who gave birth to a preterm infant vs directly given to preterm neonates. They concluded that there is insufficient evidence to conclude whether there is appreciable benefit or harm to neonates after maternal administration of probiotics either pre or postnatally. Thus, more research is needed. In this study, we chose a biological approach using an animal model of immaturity to evaluate the role of probiotics in normal gut development and physiology. We hypothesized that specific strains of probiotics given to pregnant mothers may be a means of influencing the infant microbiome to optimize intestinal development. Since both probiotic strains *Lactobacillus acidophilus* and *Bifidobacterium infantis* (LB) are well documented in the prevention of NEC in preterm infants and in experimental NEC models when given to the hosts [12, 13], we utilized a mouse model to test the effect of the administration of a combination of LB to pregnant dams from late pregnancy through lactation on intestinal outcomes and host-microbe interactions in preweaned pups. We investigated whether maternal LB exposure could modulate intestinal maturation and host–microbe interaction in the developing offsprings. We report that maternal LB administration elicited significant beneficial effects on the growth and maturation parameters of the immature intestinal mucosa; protected intestinal barrier function and modulated intestinal inflammatory responses and influenced early intestinal microbiota colonization in the offsprings.

## Methods

### Animals

This study was carried out in strict accordance with the recommendations in the Guidelines for the Care and Use of Laboratory Animals from the National Institutes of Health. All animal work was conducted under animal protocol No. 71703 and was approved by the University of Chicago Institutional Animal Care and Use Committee (IACUC). Time-dated pregnant C57/BL6J specific pathogen-free (SPF) mice were obtained from Jackson Laboratory (Bar Harbor, ME) and were kept on a 12 h light/dark cycle with access to food and water *ad libitum*. On embryonic day 15 (ED15), SPF dams were fed a 10^9^ colony forming unit (cfu) dosage each of *Lactobacillus acidophilus* and *Bifidobacterium infantis* (LB) daily until pup sacrifice and the offspring are denoted throughout results as SPF/LB. Previous studies have established that the dose range for these two strains of probiotics is 0.5-2x 10^9^ [14]. Control SPF pregnant dams were fed PBS and the offspring are denoted throughout results as SPF. Pups were delivered naturally and remained with their mothers until sacrifice. Preweaned (2-weeks-old) pups were used for this study as preweaned mouse intestinal epithelium (P14) is developmentally comparable to preterm infant intestine [15]. Besides routine monitoring of animal well-being at the facility, during the experimental periods, the dams were checked and weighed daily by a trained technician. If distress signs such as hunched back, weight loss, dystocia or lethargy appeared the animals would be euthanized per institutional guideline using CO_2_ followed by cervical dislocation.

The pups were divided in two groups and were either intraperitoneal (i.p.) injected with saline as a control or i.p. injected with IL-1β at 50 ng/g body weight to model the systemic inflammation observed in NEC patients. After four hours of IL-1β (cat# 50813285, Thermo-Fisher) treatment, mice were euthanized under isoflurane followed by cardiac puncture for blood collection, and sera were collected and stored at -80°C for further analysis. Small intestinal tissues were harvested and snap frozen on dry-ice-ethanol bath for biochemical analysis, and the terminal ileal tissue was fixed and processed for histological and immunohistochemical analysis.

### Probiotic bacteria preparation

*Lactobacillus acidophilus*, and *Bifidobacteria infantis* used were from ATCC (No. 53544 and 15697, respectively). Bacteria were first grown and expanded in MRS broth (DeMan Rogosa & Sharpe, Difco) at 37°C and 5% CO2 under anaerobic and non-agitating conditions, centrifuged (20 min, 4,500×g), and resuspended in modified Hank’s balanced saline solution (HBSS) supplemented with 0.04 M MgSO4, 0.03 M MnSO4, 1.15 M K2PO4, 0.36 M sodium acetate, 0.88 M ammonium citrate, 10% polysorbate (growth factor for *Lactobacillus* sp) and 20% dextrose. Bacteria then were propagated overnight at 37°C, 5% CO2 under nonagitating conditions to 2×109 cfu/mL.

### *In vivo* intestinal barrier function assay

Fluorescein isothiocyanate (FITC)-labeled dextran molecules (4 kDa molecular weight, Cat# 46944, Sigma) were fed to the mouse pups to monitor change in intestinal permeability in response to IL-1β challenge. In our previous studies, 4 kDa FITC-dextran produced more consistent robust responses than other MW FITC-dextran given the 2h time frame [16]. Briefly, two hours after IL-1β injection, pups were fed with 4 kDa FITC-dextran (40 mg/100 g body weight) via an orogastric tube. Two hours later, whole blood was collected in eppendorf tubes at room temperature and then spun at 8000rpm in a microcentrifuge for 10 min. The serum was used for the measurement of fluorescence intensity using a Synergy 2 Multi-fluorometer Reader by BioTek Instruments Inc (Winooski, VT). FITC-dextran flux/concentration (μg/ml) was calculated with standard curves. Experiments were performed in duplicates and higher concentrations of FITC-dextran in the serum indicated poorer barrier function.

### Cytokine assay

To monitor the systemic inflammatory responses to IL-1β challenge, sera were collected from preweaned pups and analyzed by multiplex analysis using a kit containing a panel of mouse cytokines/chemokines (MCYTOMAG-70K), which utilizes MAGPIX^®^ System in combination with Luminex^®^ xMAP^®^ technology with magnetic beads, according to the manufacturer’s instructions (EMD Millipore Corporation, Billerica, MA). Experiments were performed in triplicate. The kit enables simultaneous analysis of 12 cytokines, chemokines and interleukins in a 25μl (2x diluted) serum sample.

### Morphology, immunohistochemistry and immunofluorescence studies

Ileal tissues were obtained from the preweaned mice and were either formalin fixed or Carnoy’s solution fixed to preserve the mucus layer at the apical surface, and then paraffin embedded. Sections (5 μm) were cut and used for morphology and immunohistology. The hematoxylin/eosin (H&E) alcian blue and PAS/Alcian blue stained sections were used for morphometric analysis under a light microscope. Villus height and crypt depth were measured in the ileum of LB (n = 7) and control (n = 8) mice. Villus length and crypt depth were quantified as a mean of 50–70 well-oriented crypt-villus units from 10 high power fields (40X) per animal. The crypt-villus units were defined as a crypt sectioned parallel to the crypt-villus axis with an unbroken epithelial column extending to the villus tip. Apical mucus area of 10 measurements per section/mouse was measured in 5 mice per condition using Image J software (U. S. National Institutes of Health, Bethesda, Maryland, USA, http://imagej.nih.gov/ij/).

For Immunohistochemistry and immunofluorescence studies, ileal tissue sections from LB and control groups were deparaffined, rehydrated and then incubated with blocking solution (5% goat serum) in PBS. The tissue sections were incubated with 50 μl of the respective primary antibody solution overnight at 4°C. After washing with PBST, the sections were incubated with the respective fluorophore-conjugated secondary antibodies and then counterstained with DAPI-antifade mounting medium (P36935, Invitrogen Inc., Carlsbad, CA, USA). Imaging was performed at the University of Chicago Integrated Light Microscopy Facility. Images were captured with a Leica TCS SP8 laser scanning confocal microscope (Leica Microsystems, Inc., Buffalo Grove, IL). Imaging processing and analysis was obtained using ImageJ.

### Apoptosis analysis of small epithelial cell death

Ileal tissue sections from LB and control groups were deparaffined and rehydrated. TUNEL staining of apoptotic cell death was done using the In Situ Cell Death Detection Kit, (TMR red 12156792910 Sigma, St. Louis MO)) and the procedure was based on the manufacturer’s recommendation. A fluorescent microscope Olympus "fixed cell" DSU Spinning Disk Confocal Axi200 (OLYMPUS Corporation) was used to identify apoptotic cells.

### Antibodies

Antibodies NF-κB (pP65) (ab86299), Ki67 (ab16667), Lgr-5 (ab75732) were purchased from Abcam (Cambridge, MA). Anti-MUC2 (NB120-11197 Novus Biological Centennial, CO), Anti-ZO-1(339188), anti-OCLN (331594) and Anti-Tff3 (PA5-21081) were obtained from Invitrogen. The primary antibodies were used at 1:100 dilution except anti NF-κB (pP65) at 1:500 dilution.

Secondary Alex Fluor^®^ goat anti-rabbit IgG (H+L) (A11034) and Alex Fluor^®^ goat anti-mouse-594 (H+L) (A11032) were purchased from Invitrogen Inc. (Thermo Fisher Scientific). All the secondary Alex Fluor antibodies were applied at 1:1000 dilutions.

### 16S rRNA profiling and analysis of the fecal microbiome

Fecal pellets were collected from the colon and rectum during the tissue harvesting step and stored at -80°C until further usage. Mouse fecal DNA was extracted from these stool samples using the QIAamp Power Fecal DNA kit (Qiagen) according to the manufacturer’s instructions. Library preparation and sequencing were performed by the Next Generation Sequencing Core at the Argonne National Laboratory using the Earth Microbiome Project protocols [17] and an Illumina MiSeq. The data was demultiplexed using QIIME2. The obtained amplicon sequence variants (ASVs) were classified to the genus level by IDTAXA utilizing the default parameters and the GTDB database version 86. Each ASV was attributed to a unique species, and those that did not represent at least 0.1% of the mean total read count across all samples were removed.

### Statistics

The results from the above data are presented as mean ± SEM. Student’s t test was performed for two group comparisons. One-way ANOVA followed by Tukey’s post-*hoc* test for multiple comparison testing among four experimental groups was performed using GraphPad Prism version 6.00 for Windows (GraphPad Software, La Jolla, California, USA, www.graphpad.com). A p-value of less than 0.05 was considered statistically significant.

Statistical analysis of the microbiota compositional differences between the Control (n = 12) and LB (n = 7) groups was conducted at the species, genus, family, class and phylum taxonomic levels and all figures were generated in R. The Wilcoxon rank-sum test was applied to determine significance (p-value < 0.05), in addition to the calculation of effect sizes. An effect size > |1| was considered to be of interest. PERMANOVA, with subsequent visualization by NMDS plots, from R package vegan version 2.5.5 was used to determine significance (p-value < 0.05) on the Euclidean distance matrix. The similar B-dispersion assumption for PERMANOVA was confirmed by ANOVA.

## Results

### Safety of administration of live *Lactobacillus acidophilus* and *Bifidobacterium infantis* (LB)

All pregnant and lactating dams that received the daily probiotic mixture via gavage produced viable litters throughout the study. The dams had no sign of stress (hunched backs, difficulty moving, loss of appetite or weight loss). The litter size and body weight for the pups birthed from the control dams were comparable to the litters born from LB dams. The litter sizes varied from 5–11 (SPF) vs. 5–10 (SPF/LB) and body weights at 2-wk of age were 7.01 ± 0.19 (SPF) vs. 6.86±0.14 (SPF/LB). These results suggest that the doses and duration of these probiotic strains were well tolerated and safe for the pregnant dams and their offsprings.

### Maternal LB facilitates intestinal growth and maturation in pre-weaned mouse pups

**Fig 1:**
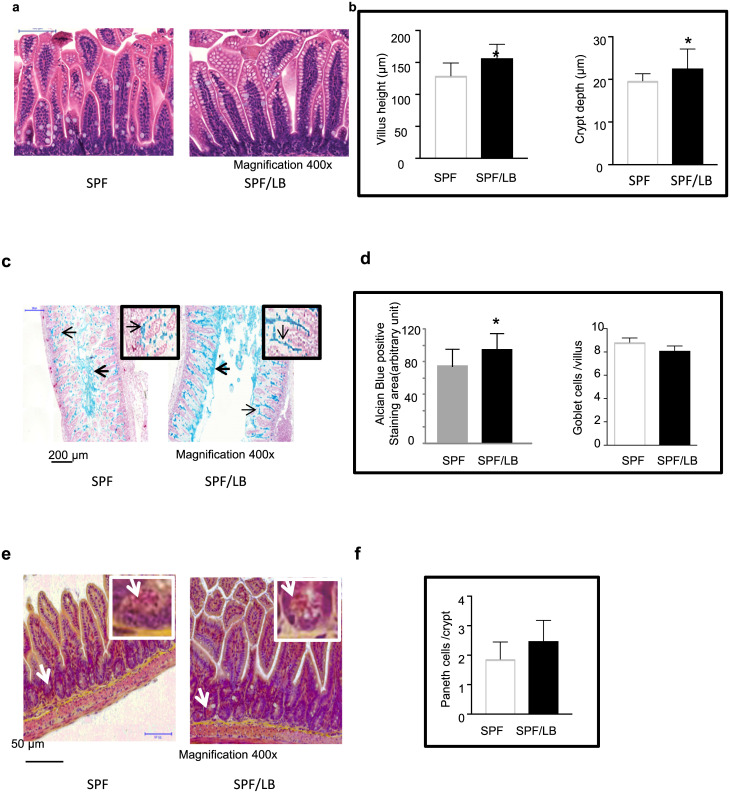
Maternal LB effects on intestinal development in 2-wk-old mouse pups. **a)** H&E staining of SPF and SPF/LB 2-wk-old mouse pup ileal tissue. **b)** Mean ileal villus height and mean crypt depth (SPF n = 8; SPF/LB n = 7). Results are presented as mean ± SEM. The student’s t-test was used to compare the groups. *p < 0.05. **c,d)** Alcian blue staining of mucus on ileal apical surface are presented. The total alcian blue stained areas were measured and quantification of mucus areas are presented as (mean±SEM, n = 4). The student’s t-test was used to compare groups. *p < 0.05, Bar = 200 μm. Alcian blue positive goblet cells were counted in 20 villi/sections and presented as (mean±SEM, n = 4). The insets represent goblet cells in the ileal villi. **e,f)** Phloxine B-tartrazine staining of intestinal Paneth cells in the crypts of ileal sections. Mean Paneth cells from 20 crypts/section were counted and presented as (mean±SEM, n = 3/group). The inset represents typical Phloxine B-tartrazine staining positive Paneth granules.

The small intestine is the major site of nutrient absorption and its surface area is important for nutrient uptake. To characterize the effect of maternal LB probiotic exposure on the morphology of the developing small intestine, we compared the preweaned mouse ileum morphology between the groups. Fig 1a represented the morphology of the villus and crypts of the ileal tissues. LB exposed pups displayed increased villus height (157.3 ± 21.0 n = 7) compared to the control pups (129.3 ± 19.9, n = 8, * p < 0.05), and crypt depth (22.6 ± 4.5 vs (19.7± 1.67), * p < 0.05, Fig 1b).

We also examined the number of goblet cells and the area of the mucus layers. The number of goblet cells and the area of the mucus layers were quantified using Paranoma software. Compared to control pups, LB promoted apical mucus formation in 2-weeks-old pups as noted by the increased alcian blue positive area (dotted line). There was no significant effect on goblet cell numbers in the ileal villi (inset representative of alcian blue positive goblet cells on the villi) (Fig 1c and 1d). Phloxine B-tartrazine staining of intestinal Paneth cells (thick arrow) were counted and shown in Fig 1e. There was no significant difference in Paneth cell numbers per crypts between two groups. The insets are representative of positive staining of Paneth cell granules (thin arrow).

**Fig 2:**
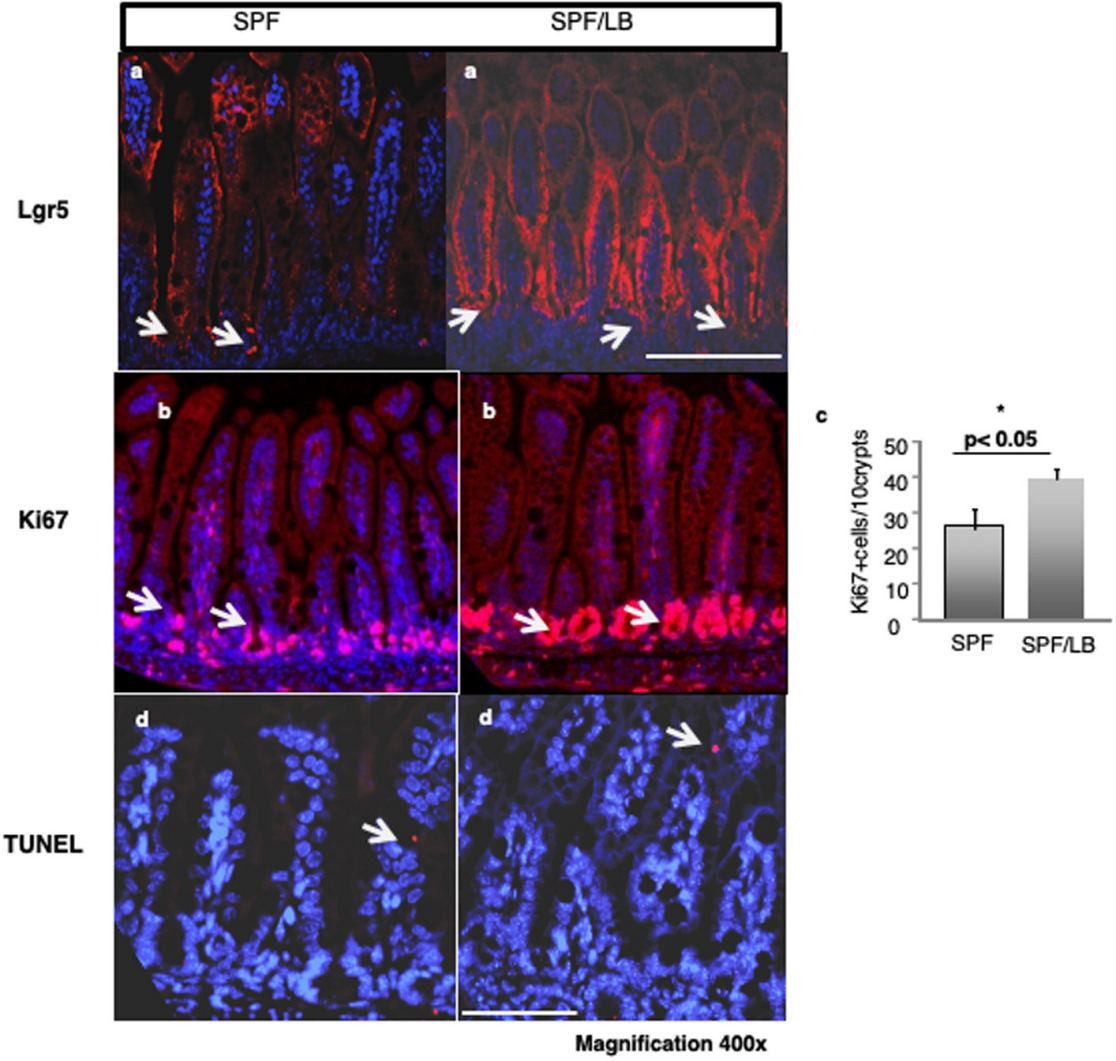
Maternal LB modulates intestinal epithelial cell proliferation in preweaned mouse pups. **a)** Immunofluorescence detection of LGR5 positive cells (red) in mouse ileal crypts of SPF and SPF/LB. Representative areas (n = 4 per group) are shown. Bar: magnification 400x; white arrows indicate positive staining in the crypts. **b)** Immunofluorescence detection of Ki-67 positive cells (red) in the crypts. Representative areas (n = 4 per group) are shown. Bar: 100 μM. magnification 400x. **c)** Quantification of Ki-67 positive cells (mean±SEM) and student’s t-test *p < 0.05. **d)** TUNEL staining for apoptosis was analyzed in mouse ileal tissue sections with or without LB (In situ Cell Death Detection Kit, Fluorescein; Roche Diagnostics). Representative areas (n = 3 per group) are shown. Bar: magnification 400x.

To understand the cellular basis for the increase in villus height and crypt depth we next examined the expression of the intestinal stem cell and transient proliferating cell marker Lgr5 in preweaned mouse ileum. As shown in Fig 2a, compared to the control ileum, LB ileum displayed higher numbers of Lgr5 positive cells in the crypts and higher levels of Lgr5 expression along the villus/crypt axis. Next we examined the rate of intestinal progenitor cell proliferation by measuring the number of Ki-67 positive (proliferating) cells in the ileal crypts and TUNEL assay for the rate of cell loss due to the apoptotic death using immunofluorescence staining. As shown in Fig 2b, the number of proliferating cells was significantly higher in pups exposed to maternal LB compared to control pups (39.5 ± 2.7 vs. 27.7 ±5.1, p < 0.05). As shown in Fig 2d, there was no difference in basal apoptosis between SPF control and LB exposed pups.

### Maternal LB exposure rescued IL-1β induced gut barrier dysfunction in preweaned pups

**Fig 3:**
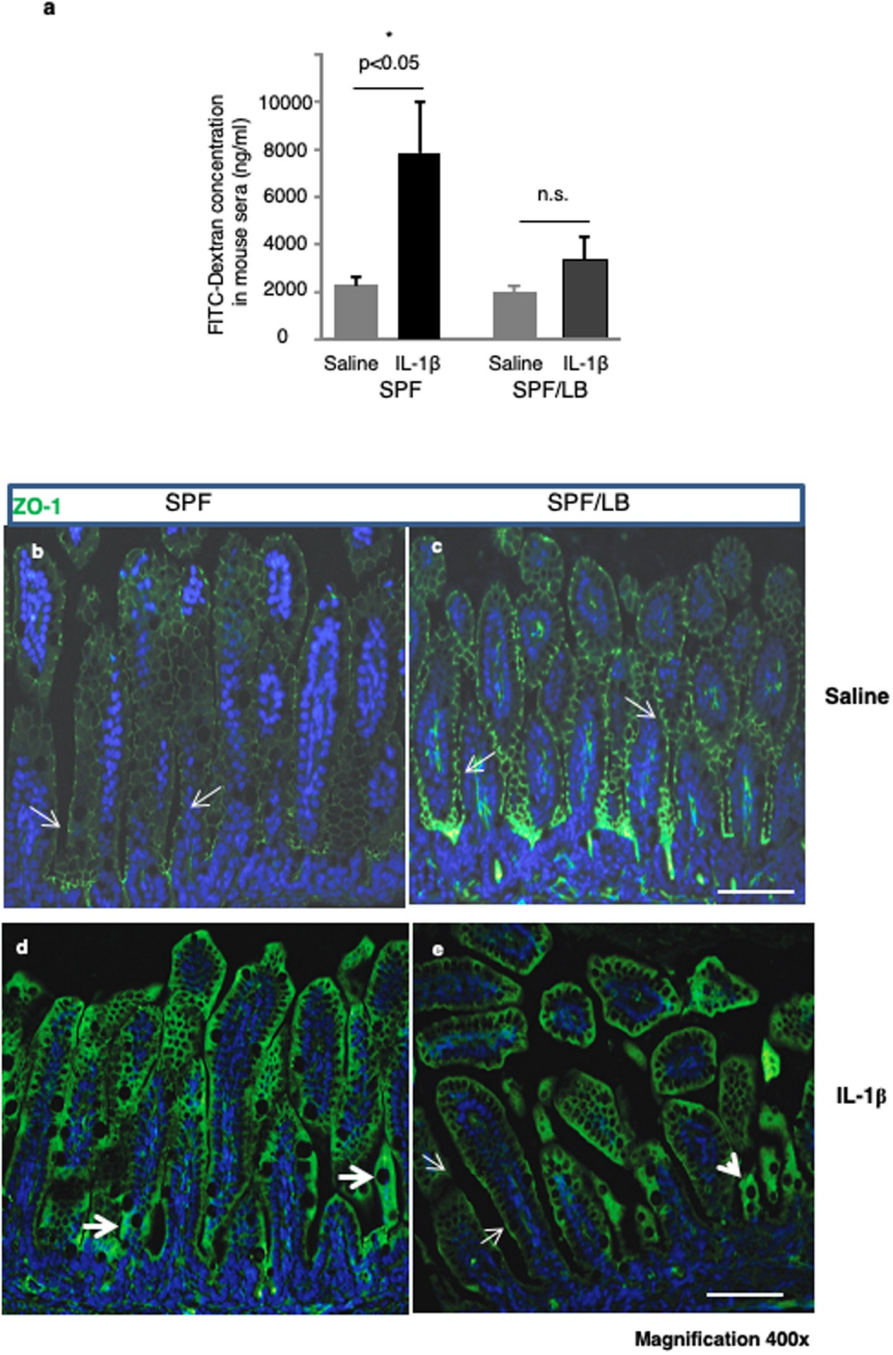
Maternal LB exposure rescued IL-1β induced gut barrier dysfunction in preweaned pups. **a)** IL-1β induced elevation of serum FITC-dextran translocation in SPF (n = 5) and SPF/LB (n = 6) was measured and presented as mean ± SEM. One-way ANOVA and Tukey’s post-hoc test was used and *p < 0.05. **b)** Immunofluorescence detection of ZO-1 molecules (green) in the SPF mouse ileum. **c)** Immunofluorescence detection of ZO-1 molecules (green) in the SPF/LB mouse ileum Representative areas (n = 3 per group) are shown Bar: 100 μM, magnification 400x. **d)** IL-1β stimulation induced disruption of tight junction (ZO-1) in SPF pups. **e)** LB protected tight junction integrity in SPF/LB pups. Immunofluorescence detection of ZO-1 translocation is indicated by white block arrows. (n = 3 per group, magnification 400x).

Systemic inflammation can disrupt gut barrier integrity. To determine if maternal LB-exposure elicited cytoprotective effects against IL-1β insult, we evaluated intestinal barrier function by measuring the rate of transmucosal transport of fluorescent FITC-dextran (4 kDa) to the bloodstream. As shown in Fig 3a, systemic administration of IL-1β significantly increased ileal permeability in control SPF preweaned pups (SPF: Saline 2263.3±403.8 vs IL-1β: 7913.9.6±2132.2 ng/ml, n = 8.8, p < 0.05). In comparison maternal LB treated pups had significantly decreased blood FITC-dextran concentrations after IL-1β administration compared to the control groups of pups (SPF/LB: Saline1936.5 ±356.6 ng/ml vs. IL-1β 3339.3 ± 936.4, n = 5, 6), indicating that maternal LB administration protected the immature host against excessive systemic inflammation and preserved mucosal barrier integrity.

To further evaluate the effect of maternal LB administration on intestinal mucosal integrity, we examined the tight junction protein Zonula Occludens-1 (ZO-1) expression and localization on the mucosal surface in preweaned mouse ileum after systemic IL-1β challenge. As shown in Fig 3 SPF pups exhibited cell surface ZO-1 expression with punctate dense structures indicating tight junction formation on the apical surfaces (Fig 3b). LB further upregulated apical surface ZO-1 expression (Fig 3c). Systemic IL-1β challenge induced tight junction disruption with a loss of apical surface ZO-1 integrity and intracellular translocation of ZO-1 molecules (Fig 3d, block arrows) in ileal mucosa. Maternal LB exposure partially protected tight junction integrity as shown by the increased preservation of cell surface ZO-1 expression indicated by the thin white arrow in Fig 3e.

### Maternal LB modulates IL-1β-induced goblet cell Tff3 and MUC2 production in response to inflammatory stress in pre-weaned mice

**Fig 4:**
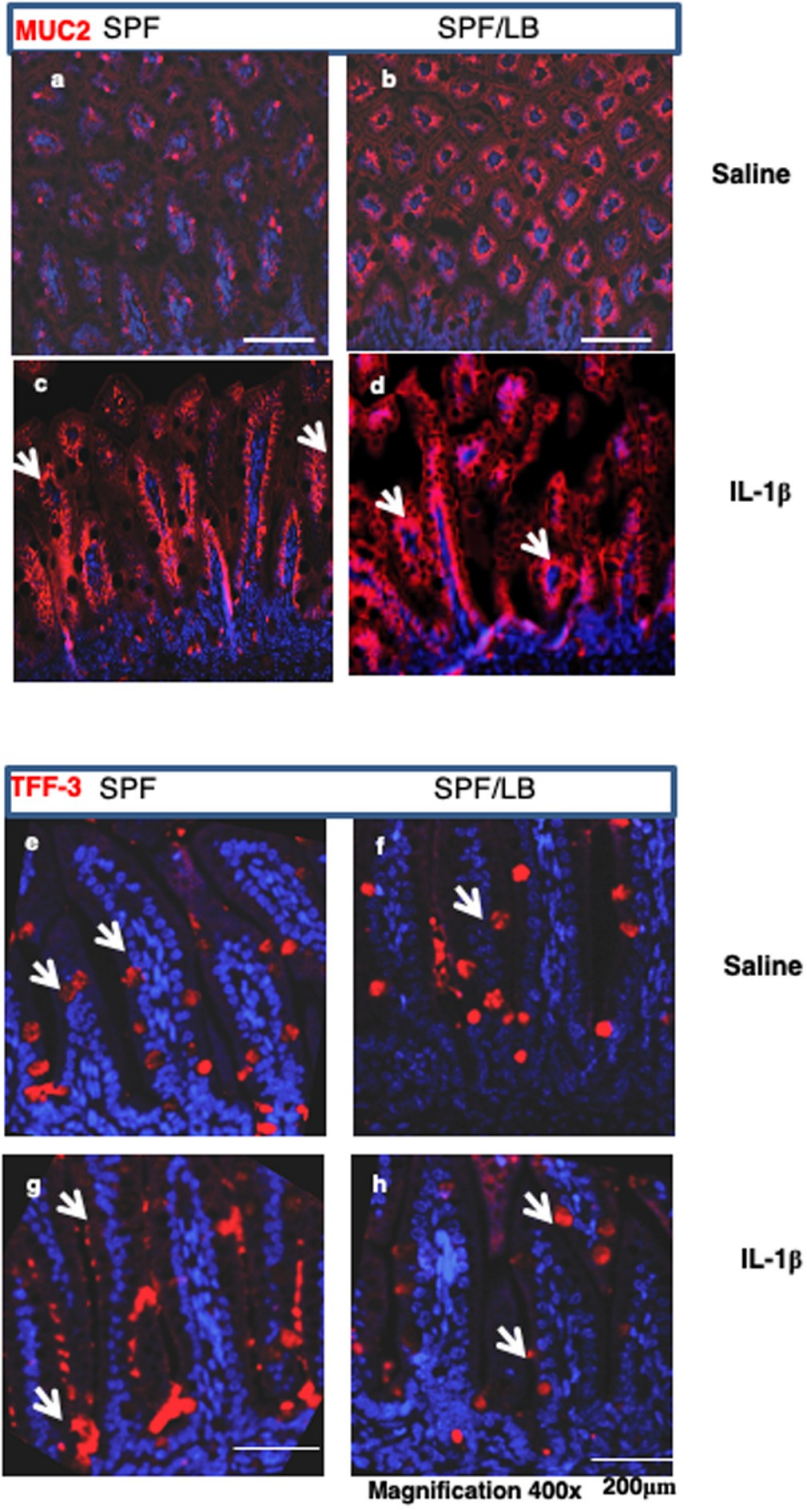
Maternal LB modulates IL-1β-induced goblet cell Tff3 and MUC2 response to inflammatory stress in pre-weaned mice. **a,b)** Immunofluorescence detection of MUC2 molecules (red) in the mouse goblet cells in SPF (a) vs SPF/LB pups (b). Representative areas (n = 3 per group) are shown Bar 100 μM, magnification 400x. **c,d)** Representative areas of MUC2 exclusion from goblet cells in response to IL-1β stimulation in SPF (c) or SPF/LB (d) pups are shown; magnification 400x. **e,f)** Representative area of immunofluorescence staining of TFF3 molecules (red) in the goblet cells. Left: SPF (e), and Right: SPF/LB (f) n = 3/per group) group, **g,h)**. IL-1β induced TFF3 (red) secretion from goblet cells was detected by immunofluorescence staining. Representative areas were shown: Left: SPF (**g**), and Right: SPF/LB (**h**) (n = 3/per group) (magnification 400x).

The mucus layer in the intestine affects several aspects of intestinal biology, encompassing physical protection, chemical protection, immunomodulation and growth, thus playing a role in maintaining mucosal homeostasis. As shown in Fig 4, compared to SPF pups in Fig 4a, LB enhanced baseline MUC2 expression in the preweaned ileal mucosa (Fig 4b). While, IL-1β challenge induced expression of MUC2 in the goblet cells in both SPF and SPF/LB pups, there is a depletion of intracellular MUC2 in SPF ileal goblet cells, an indication of goblet cell response to stress (Fig 4c). However, maternal LB modulated the stress response of the goblet cells and preserved mucosal surface MUC2 expression in SPF/LB pups after IL-1β challenge (Fig 4d block arrow).

Intestinal trefoil factor 3 (Tff3), a mucin-associated secretory molecule produced by goblet cells, promotes intestinal epithelial wound healing and modulates epithelial cell adhesion, migration and survival [18]. We next examined Tff3 expression and function in the preweaned mouse intestine in response to IL-1β stimulation. As shown in Fig 4, both SPF (Fig 4e) and SPF/LB (Fig 4f) pups expressed comparable levels of TFF3 peptides in the goblet cells at baseline. Upon IL-1β induced inflammatory stimulation, in the SPF intestine, a depletion of Tff3 staining in the goblet cells and aggregation of Tff3 on the mucosal surface was observed (Fig 4g, white arrows). In contrast, there was no significant depletion of Tff3 in the goblet cells and less aggregates on the epithelial surface with maternal LB exposure (Fig 4h), further indicating that LB can modulate intestinal mucosal stress response to inflammatory challenge.

### Maternal LB exposure significantly attenuated postnatal IL-1β induced systemic inflammation in the offspring

**Fig 5:**
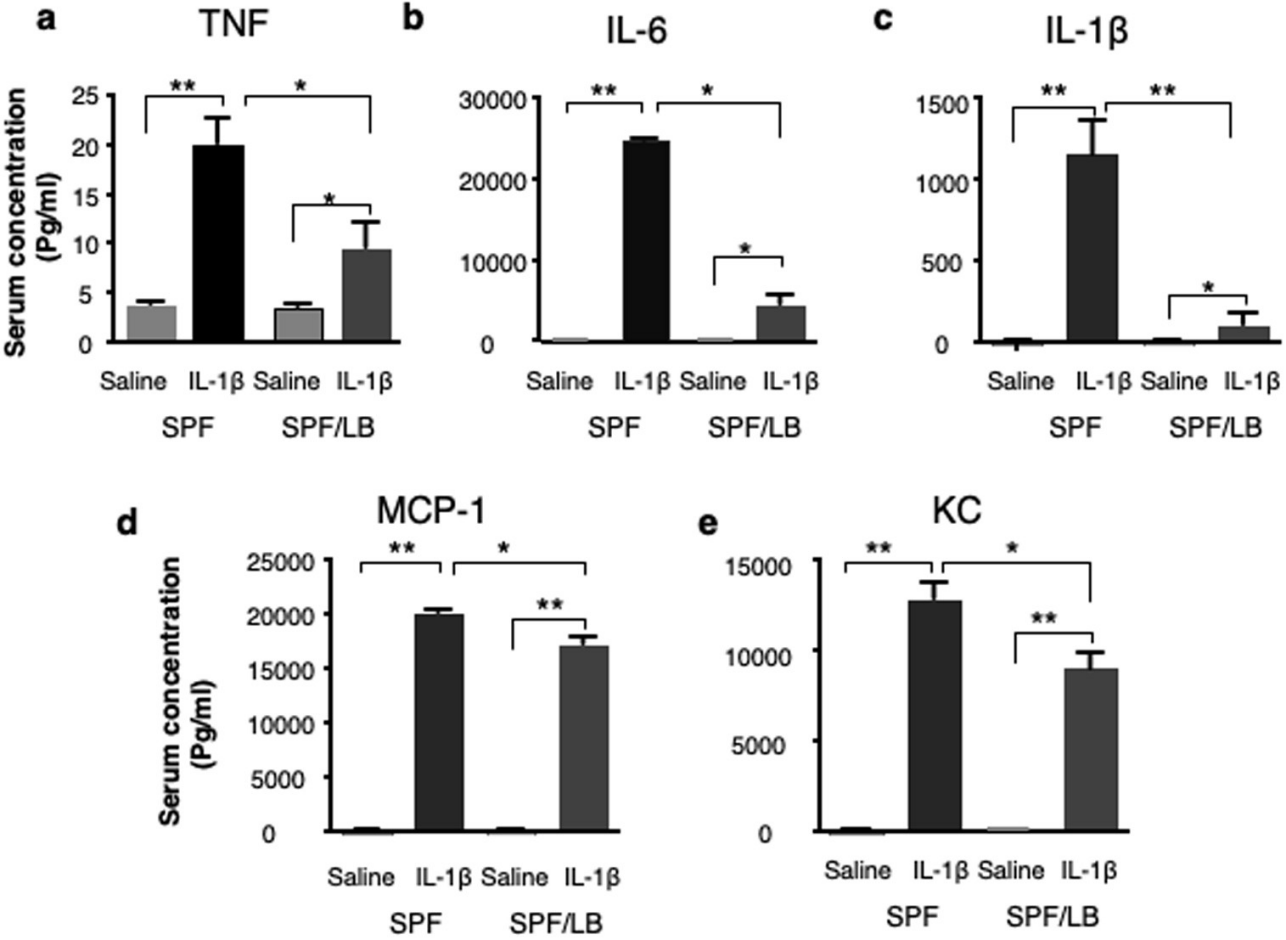
Maternal LB modulates inflammatory cytokine secretion in response to IL-1β in preweaned pups. Serum concentrations of inflammatory cytokines were quantified using a multiplex immunoassay of inflammatory cytokines in preweaned pups (n = 4/per group). (**a**) TNF, (b) IL-6, (**c**) IL-1β, (**d**) MCP-1 and (**e**) KC are presented as mean ± SEM. One-way ANOVA and Tukey’s post-hoc test were used to detect differences among the groups. Bars denote a significant difference between experimental groups (* p < 0.05, **p < 0.01).

Preterm infants have an excessive inflammatory response to stressors [7]. To examine if maternal administration of LB modulates the response to systemic inflammation in the immature offspring, we challenged preweaned mouse pups with systemic IL-1β exposure. As shown in Fig 5, IL-1β elicited a strong systemic inflammatory response resulting in elevated concentrations of serum TNFα, IL-6, IL-1β, MCP-1 and KC in the 2-weeks-old mice. Maternal LB exposure significantly attenuated the IL-1β-elicited inflammatory responses in these pups compared to the unexposed control groups (p < 0.05, n = 4).

### Maternal LB exposure modulated IL-1β induced NF-κB activation

**Fig 6:**
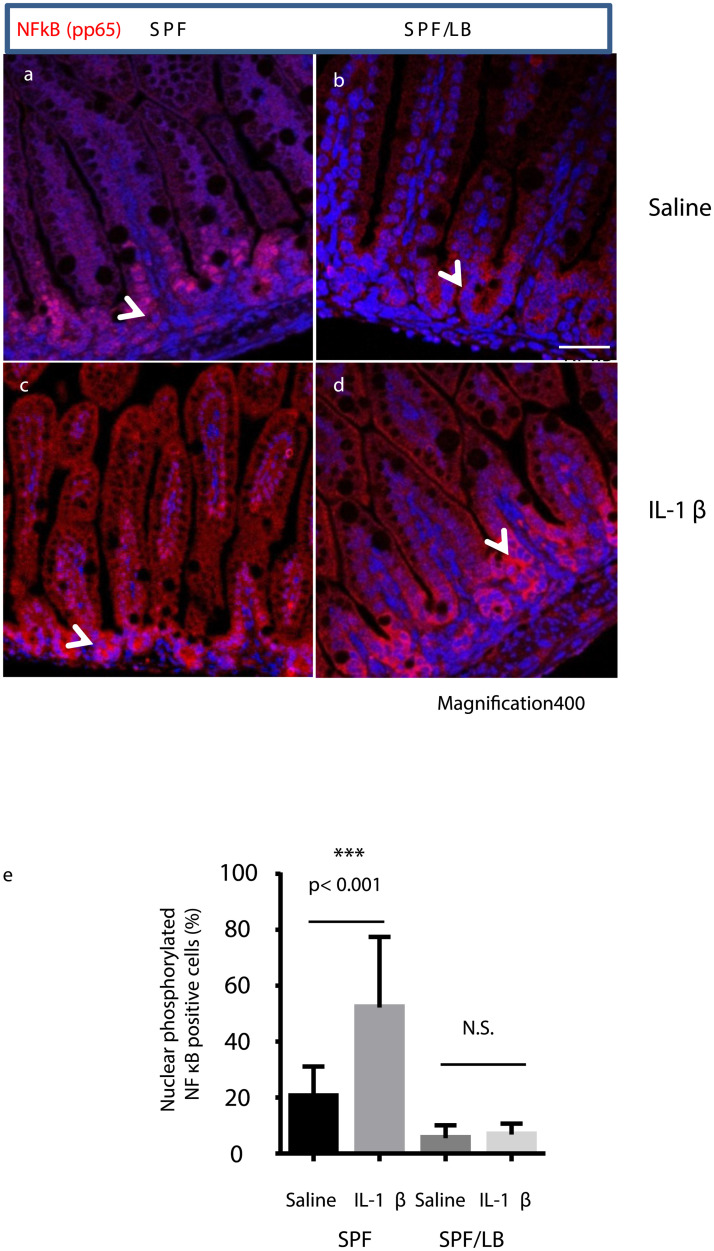
Maternal LB modulates NF-κB activation in preweaned pups. IL-1β induced NF-κB activation was analyzed by immunofluorescence staining of the ileum. Representative areas (n = 3 per group) (magnification 400x); white arrows indicate positive staining of the phosphorylated NF-κB p65 subunit (pp65). Basel level of pp65 in SPF (a) and SPF/LB (b) are shown. IL-1β induced pp65 nuclear translocation are presented in SPF (c) and SPF/LB (d). Percent of pp65 positive nuclei were quantified by the ImmunoRatio tool, mean % ± SEM. A one-way ANOVA and Tukey’s post-hoc test were used to detect differences among the groups, **p < 0.01.

The transcription factor NF-κB, a master regulator of innate and adaptive immune functions, serves as a pivotal mediator of inflammatory responses and has increased activation in the immature gut [19]. We therefore investigated whether maternal LB probiotic exposure modulated NF-κB activation in the mouse pups. Immunohistochemistry analysis was performed to examine the nuclear localization of phosphorylated p65 (pp65) as an indicator of NF-κB activation. As shown in Fig 6, in SPF control pups, IL-1β exposure induced massive nuclear translocation of NF-κB as represented by the positive staining of pp65 in the crypt cells, which indicates an acute intestinal response to inflammatory stimuli (Fig 6c vs 6a. In contrast, the maternal LB exposed pups exhibited higher level of resting NF-κB (Fig 6b), as indicated by the perinuclear staining of the p65 subunit in the crypt cells. Upon IL-1β challenge, significant amount of NF-κB remained in the resting state for these pups, as represented by the persistent perinuclear pp65 staining in the majority of cells (Fig 6d). The numbers of phosphorylated p65 positive nuclei in the ileal tissues were quantified using the Image J. The difference in the presence of nuclear pp65 was quantified and presented in Fig 6e.

### The effects of maternal LB exposure on the intestinal microbiota composition of the offspring

**Fig 7:**
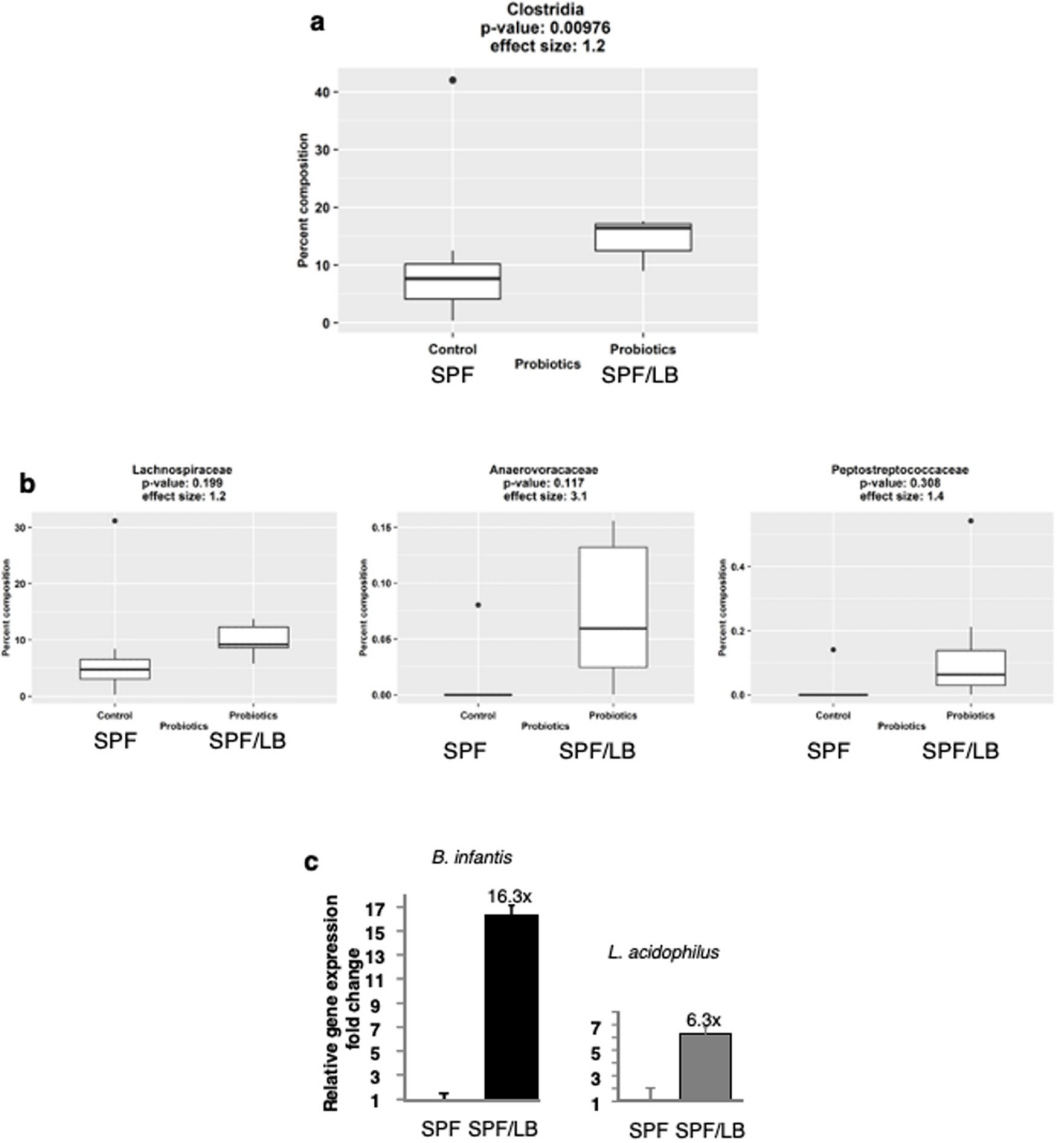
Maternal LB modulates the intestinal microbiota in preweaned mouse. **a)** The relative abundance of Clostridia class was found to be significantly different between groups (SPF n = 12, SPF/LB n = 7) by the Wilcoxon rank-sum test; **b)** three Clostridial families with large effect sizes between groups, **c)** Relative abundance of *B*. *infantis* and *L*. *acidophilus* in SPF/LB pup fecal DNA compared to that of control SPF pups (n = 3).

Fecal samples from 2 weeks of life were evaluated. We found that maternal LB administration did not result in significant changes in overall alpha diversity (richness, Shannon diversity and Simpson diversity, S1A Fig). There were no significant differences in overall beta diversity at the taxonomic levels of family and lower S1B Fig. However, after examining the individual taxa, the class Clostridia was significantly increased in abundance (p < 0.01; effect size = 1.2) in SPF/LB pups (Fig 7a). Although no further significant differences were found at the lower taxonomic levels for this time point, we examined the bacterial families that had high effect sizes between the SPF and SPF/LB pups to determine which families were most contributing to the observed change in abundance of Clostridia. We found that Lachnospiraceae (effect size = 1.2), Anaerovoracaceae (effect size = 3.1) and Peptostreptococcaceae (effect size = 1.4) were the major contributors (Fig 7b). In order to verify if the maternal administration of LB influenced the abundance of *L*. *acidophilus* and/or *B*. *infantis* in the pups, we used the species-specific primers *BI-F5’ GGG TGG TAA TGC CGG ATG 3’; BI-R 5’CCA CCG TTA CAC CGG GAA 3’*. *LA-F5’ GGA AAC AG A TGC TAA TAC CG 3’; LA-R 5’ CAC CGC TAC ACA TGG AG 3’* for analysis via real-time PCR. There was a sixteen- and six- fold difference in *B*. *infantis* and *L*. *acidophilus* expression respectively in LB exposed pup fecal DNA compared to that of control pups (n = 3) (Fig 7c).

## Discussion

NEC is a complex, multi-factorial disease. The primary predisposing factor for NEC is prematurity, which is associated with 1) immature mucosal barrier development, 2) altered barrier responses associated with increased susceptibility to inflammation and loss of epithelial integrity; and 3) abnormal intestinal microbiota patterns [20, 21]. Currently, approaches to prevent NEC have become a focus of research efforts. One of these approaches is to manipulate the intestinal microbiota by bolstering the growth of beneficial microbes. Probiotics are one potential way to achieve this aim [22, 23]. However, many regulators, researchers and clinicians are reluctant to adopt routine administration of probiotics in high risk infants owing to concerns about the quality and efficacy of available probiotic products. In the field of early microbial interactions and infant disease prevention studies, evidence indicates that maternal probiotic supplementation during pregnancy and breastfeeding could be effective in reducing the risk of immune/inflammatory and metabolic diseases in both mother and infants [24]. To our knowledge, there is only one published small clinical trial on the efficacy of probiotics administered to the mother in the primary prevention of NEC in preterm infants [25]. Benor et al reported that maternal supplementation with *Lactobacillus acidophilus* and *Bifidobacteria lactis* may decrease the incidence of NEC in breastfed very low birth weight infants [25]. Though the study size was too small to draw conclusions, it provides a rationale for the study of maternal probiotic supplementation on the offsprings’ intestinal mucosal barrier development.

In this study, we examined the effects of the specific probiotics *L*. *acidophilus* and *B*. *infantis* (LB) administered to pregnant mouse dams on three hallmarks of NEC: prematurity-associated intestinal barrier dysfunction, dysregulated inflammatory responses and microbial dysbiosis in the preweaned offsprings. The rationale for the selection of LB in our model is based on the known features of *L*. *acidophilus* (LA) and *B*. *infantis* (BI) that are relevant to preterm infant NEC reviewed in Underwood [14]. LA has direct anti-inflammatory effects by inhibiting the induction of nuclear factor-κB (NF-κB) and IL8 [26]. BI is a dominant gut microbe in full term infants due to its capacity to utilize the full range of human milk oligosaccharides (HMOs) and increased BI numbers in the feces are associated with improved growth in infants [27]. Moreover, BI has anti-inflammatory properties in both *in vivo* and *in vitro* studies. These effects include decreased incidence and severity of NEC via inhibition of inflammatory factors including IL6, IL8, TNFα, and IL23 [28].

Here, we show that maternal LB administration during pregnancy and lactation promotes intestinal development, improves small intestinal barrier function and decreases inflammatory responses of the preweaned pups. Specifically, we demonstrate that LB exposed mouse gut is more resistant to IL-1β induced barrier dysfunction by maintaining high production of the tight junction related protein ZO-1 and by preserving tight junction integrity as evidenced by decreased permeability as shown in Fig 3. We also found specific effects of LB on goblet cells. The goblet cells and the mucus layers are crucial components of the intestinal barrier that impede direct microbial-epithelial binding and enhance removal of adherent bacteria [29]. In contrast, an immature mucin layer (frequently seen in preterm infant intestines) might lead to increased intestinal permeability and enhanced bacterial adherence, potentially breaching the intestinal epithelial barrier and increasing susceptibility to injury [30, 31]. Since preweaned pups and preterm infants have an immature immune system, this mucosal barrier integrity is particularly essential for their well-being. Consistent with recent studies [32, 33], we show in this study that maternal LB supplementation promotes the functional maturation of goblet cells indicated by enhanced production and secretion of MUC2 proteins resulting in a thicker mucus layer along the apical surface of the ileum and prevention of the loss of Tff3 and MUC2 molecules in the goblet cells of the preweaned mouse ileum after inflammatory insult (Fig 4). These results suggest that maternal LB can protect functional goblet cell populations and prevent tight junction dysfunction associated with immature intestinal inflammatory responses in mouse pups. Maternal administration of LB also led to the downregulation of systemic inflammatory cytokine production resulted from downregulation of nuclear NF-κB activation after inflammatory insult. This could be an important cellular mechanism of LB maintaining the homeostasis of intestinal mucosa.

An unexpected finding was that maternal LB increases not only pup levels of *Lactobacillus* and *Bifidobacterium*, but also influences the abundance of specific families of Clostridia and the bacterial family Lachnospiraceae in particular. It has been observed that preterm infants display coordinated patterns in the succession of their gut microbiota, and that altered successional patterns representative of a delayed maturation occur in infants that develop NEC [34, 35]. Members of the Firmicutes phylum, such as the families Staphylococcaceae, Clostridiaceae, and Lachnospiraceae, are more numerous in breastfed infants than in formula-fed, full-term infants [36]. The Lachnospiraceae family contains many butyrate producers [37] and butyrate has been found to promote intestinal epithelial cell proliferation, differentiation and formation of tight junctions [38]. This is consistent with our observations of improved intestinal development. This increase in Clostridia abundance may be explained by their propensity for cross-feeding on lactate. *Bifidobacterium infantis* can digest most milk oligosaccharides and thus provide an increased food source for Clostridia family. Recent studies have shown that supplementation of probiotics particularly *Bifidobacteria* and *Lactobacillus* in human and rodents increases the relative abundance of lactate-utilizing species within the microbiota [39]. Certain Clostridiaceae and Lachnospiraceae have the ability to grow in the presence of lactate and acetate to produce butyrate [40]. Besides lactate, cross-feeding of polysaccharide breakdown products released by *Bifidobacteria* is another route for the increased abundance of butyrate producing bacteria including Clostridiaceae, Lachnospiraceae, and Ruminococcaceae [41].

In conclusion, our findings suggest that maternal probiotic administration may be a safe and effective way to manipulate the immature host microbiota and foster specific intestinal homeostatic phenotypes. This is potentially a safe alternative therapy for modulating developmental processes to prevent prematurity-associated inflammatory neonatal disorders such as NEC.

## Supporting information

S1 Fig **a)**. Maternal LB supplementation had no effect on the richness, Shannon diversity and Simpson diversity of the intestinal microbiota of 2-weeks-old offspring. Stool samples were collected from SPF and SPF/LB mouse pups. 16S rRNA sequencing was performed and analyzed. Statistical significance was then determined by the Wilcoxon rank-sum test. **b)**. Differences in overall beta diversity of the intestinal microbiota between SPF and SPF/LB preweaned pups. Although statistical significance was found at the taxonomic levels of family, genus and species by a PERMANOVA, variability was high, as shown by the statistical ellipses (multivariate t-distribution) in the NMDS plots (indicated stress values verify great representation in reduced dimensions).
(TIFF)
