# Supplementary figures and images for: Maternal administration of probiotics promotes gut development in mouse offsprings
Source: PLoS One. 2020 Aug 7;15(8):e0237182. doi: 10.1371/journal.pone.0237182 (PMC7413491; doi:10.1371/journal.pone.0237182)

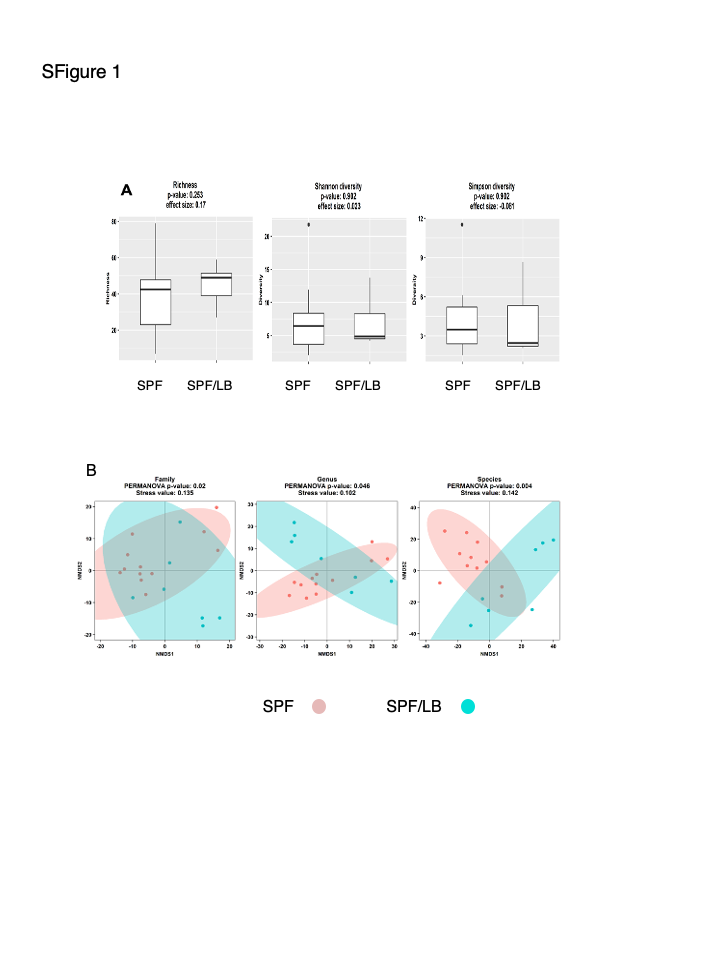

Supplement: S1 Fig — a). Maternal LB supplementation had no effect on the richness, Shannon diversity and Simpson diversity of the intestinal microbiota of 2-weeks-old offspring. Stool samples were collected from SPF and SPF/LB mouse pups. 16S rRNA sequencing was performed and analyzed. Statistical significance was then determined by the Wilcoxon rank-sum test. b). Differences in overall beta diversity of the intestinal microbiota between SPF and SPF/LB preweaned pups. Although statistical significance was found at the taxonomic levels of family, genus and species by a PERMANOVA, variability was high, as shown by the statistical ellipses (multivariate t-distribution) in the NMDS plots (indicated stress values verify great representation in reduced dimensions). (TIFF) [file pone.0237182.s001.tiff]
